# An Analysis of the Anomalous Fissure of the Ligamentum Teres Hepatis: A Morphological Perspective in the North Indian Population

**DOI:** 10.7759/cureus.58984

**Published:** 2024-04-25

**Authors:** Heena Singh, Raghvendra Singh, Rohit K Singh, Garima Sehgal, Rakesh K Dewan

**Affiliations:** 1 Anatomy, King George's Medical University, Lucknow, IND; 2 Forensic Medicine & Toxicology, Era's Lucknow Medical College and Hospital, Lucknow, IND; 3 Medicine, King George's Medical University, Lucknow, IND

**Keywords:** pons hepatis, variant fissure, visceral, tunnel, ligamentum teres hepatis

## Abstract

Background

The liver, being the largest internal organ of the body shows a variety of gross morphological variations about lobes, fissures and processes which may be clinically significant. Among various anatomical variations, the most found is the variant fissure for ligamentum teres hepatis. The present study was done to classify, review, compare and discuss the literature for anomalies in fissures for ligamentum teres hepatis.

Methods

A total of 100 formalin-preserved human livers were obtained from the Department of Anatomy of King George's Medical University, Lucknow, and studied for one year.

Result

In our study, 15% of the liver showed morphological variations in fissures for ligamentum teres hepatis. These were classified into four types. In type I (2%), the fissure was converted into a tunnel by pons hepatis. In type II (3%), there was an incomplete fissure for ligamentum teres hepatis extending into the diaphragmatic surface. In type III (4%), there was an incomplete fissure for ligamentum teres hepatis present only on the visceral surface. In type IV (6%), the fissure was covered by a thin membrane.

Conclusion

In this study of the North Indian population, 15% of liver have gross morphological variations. So thorough anatomical knowledge of the existence of variant or abnormal surface features on the liver is imperative to understanding the underlying pathology for radiologists and surgeons so that a favorable outcome can be achieved.

## Introduction

The liver resides predominantly in the right hypochondrium and epigastrium, with its reach extending into the left hypochondrium.. The visceral surface of the liver shows four constant structures - fissure for the ligamentum teres hepatis, a fissure for the ligamentum venosum, a groove for the inferior vena cava (IVC), a fossa for the gall bladder, and the porta hepatis. These divide the liver inferiorly into right, left, caudate, and quadrate lobes [1].

The fissure for the ligamentum teres emerges from the invagination of the ligamentum teres hepatis on the visceral surface of the liver, situated between the left lobe and the quadrate lobe [1]. The ligamentum teres hepatis is a vestige from embryonic development, originating from the obliterated left umbilical vein that links the placenta to the left portal vein in fetal stages. Anatomists and radiologists rely on these significant fissures and ligaments as crucial landmarks to decipher lobar anatomy and pinpoint liver lesions [2].

Until now, the ligamentum teres hepatis has been predominantly examined purely as an anatomical and embryological structure. However, there has been a recent exploration by surgeons and clinicians regarding the potential utilization of the ligamentum teres as a patch in cases involving perforated duodenal and peptic ulcers [3]. Additionally, the necrosis or abscess of the ligament, although rare, poses significant challenges in preoperative diagnosis. Hepatic imaging conducted for acute abdominal cases may lead to misinterpretation, potentially resembling other prevalent causes such as gall bladder masses or pseudopancreatic cysts [4]. Hence, it is imperative for both operating surgeons and radiologists to possess a thorough understanding of the anatomy of this structure and its commonly encountered variations. Moreover, gastroenterological surgeons utilize the fissure as a pivotal landmark during liver resections, while the ligamentum teres hepatis serves as a grasp for liver manipulation in laparoscopic liver resections [2]. Several studies have delved into the gross anatomical variations of the liver, yet scant literature exists regarding variations in the fissure for ligamentum teres [5-10]. In light of this gap, the present study endeavors to investigate different variations and their prevalence in the umbilical fissure for the ligamentum teres hepatis.

## Materials and methods

This was a retrospective study. A total of 100 formalin-fixed cadaveric livers obtained from routine dissections of donated bodies at the Department of Anatomy of King George's Medical University, Lucknow, were incorporated for the study. The donors' age ranged from 50 to 80 years. This institute serves as a major tertiary center catering to a sizable population from northern India. The study spanned over one year, from September 2022 to August 2023. The liver specimens with abnormal surface structure or size, as well as damaged livers, were excluded from the study. Each liver underwent an examination under bright daylight to identify the fissure for the ligamentum teres. Photographs of livers exhibiting notable anatomical variations in the fissures for the ligamentum teres hepatis were taken and an effort was undertaken to classify these variances based on prevalent patterns and their frequency of occurrence. Ethical Approval for this article was provided by the Institutional Ethical Committee (Registration number: ECR/262/Inst/UP/2013/RR-19) via reference code 127th ECM IIA/P4.

## Results

A total of 15 out of 100 (15%) livers showed different types of variations in fissures for ligamentum teres hepatis. We devised a classification based on our observation of variations following naked eye examination of the fissure and classified it into four types:

Type I: fissure converted into the tunnel (absent fissure)

**Figure 1:**
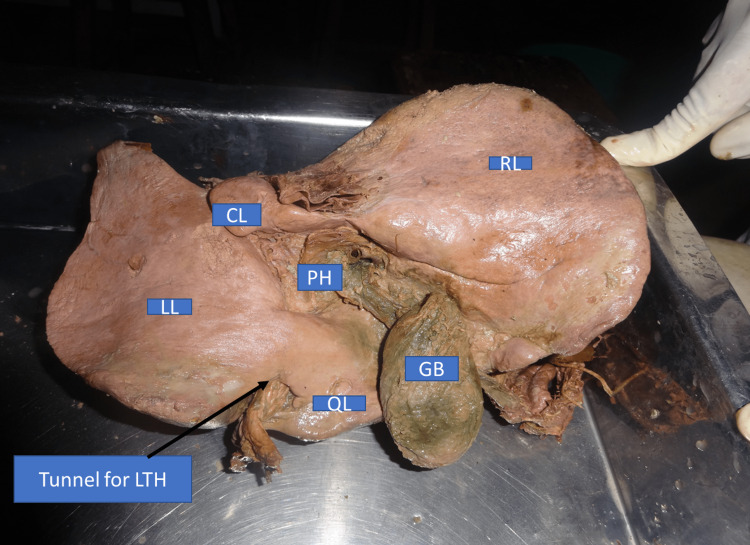
Liver with tunnel for ligamentum teres hepatis (inferior view) RL - right lobe; LL - left lobe; QL - quadrate lobe; CL- caudate lobe; GB - gall bladder; PH - porta hepatis; LTH - ligamentum teres hepatis

In this type of anatomical variant, a bridge of tissue (pons hepatis) was extending between the left lobe and quadrate lobe. Because of the presence of this bridge fissure was converted into a tunnel. However, the fissure continued up to the left end of porta hepatis and ligamentum teres hepatis entered the liver through this tunnel. This type of anatomical variation in fissure was present in 2% of liver specimens (Figure 1, Table 1).

**Table 1:** Percentage of different type of variations in fissure for ligamentum teres hepatis Type I - fissure converted into the tunnel (absent fissure); type II - Incomplete fissure for ligamentum teres hepatis extending into the anterior surface; type III - incomplete fissure for ligamentum teres hepatis present only on the visceral surface; type IV - fissure covered by a thin membrane

	No of case (15)	Percentage (out of total specimens)
Type I	2	2%
Type II	3	3%
Type III	4	5%
Type IV	6	6%

Type II: incomplete fissure for ligamentum teres hepatis extending into the anterior surface

**Figure 2:**
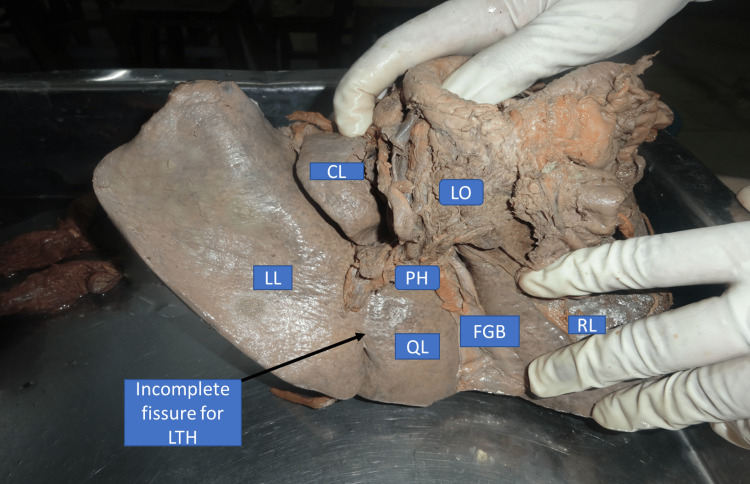
Liver with incomplete fissure for ligamentum teres hepatis extending into anterior surface (inferior view) RL - right lobe; LL - left lobe; QL - quadrate lobe; CL - caudate lobe; FGB - fossa for gall bladder; LO - lesser omentum; PH - porta hepatis; LTH - ligamentum teres hepatis

In this type of anatomical variant, a bridge of tissue connects the left lobe and quadrate lobe. However, this fissure was incomplete on the visceral surface as it did not extend up to the left end of the porta hepatis. This fissure continued into the inferior border upto the diaphragmatic surface with ligamentum teres hepatis entering the liver through the diaphragmatic surface. This type of anatomical variantion in fissure was present in 3% of liver specimens (Figure 2, Table 1).

Type III: Incomplete fissure for ligamentum teres hepatis present only on the visceral surface

**Figure 3:**
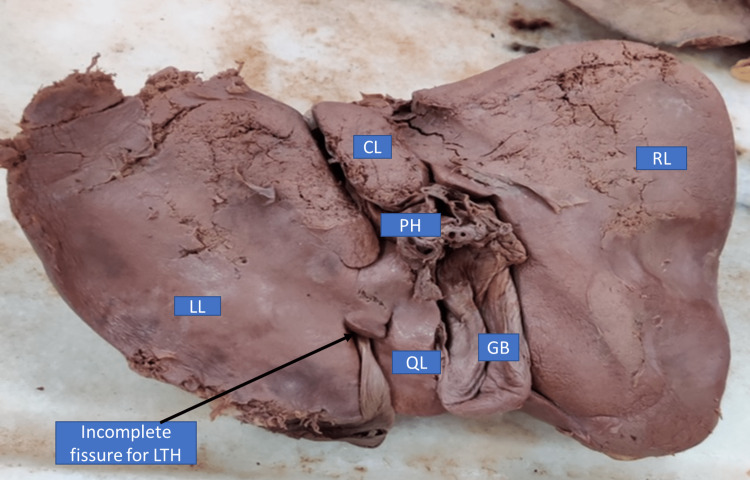
Liver with an incomplete fissure for ligamentum teres hepatis present only on posterior surface (inferior view) RL - right lobe, LL - left lobe; QL - quadrate lobe; CL - caudate lobe; GB - gall bladder; PH - porta hepatis; LTH - ligamentum teres hepatis

In this type of anatomical variant, a bridge of tissue connects the left lobe with the quadrate lobe. This fissure was incomplete, present only on the visceral surface and did not extend up to the left end of porta hepatis as in type II. However, ligamentum teres entered the fissure through the visceral surface. This type of anatomical variantion in fissures was present in 4% of liver specimens (Figure 3, Table 1).

Type IV: fissure covered by a thin membrane

**Figure 4:**
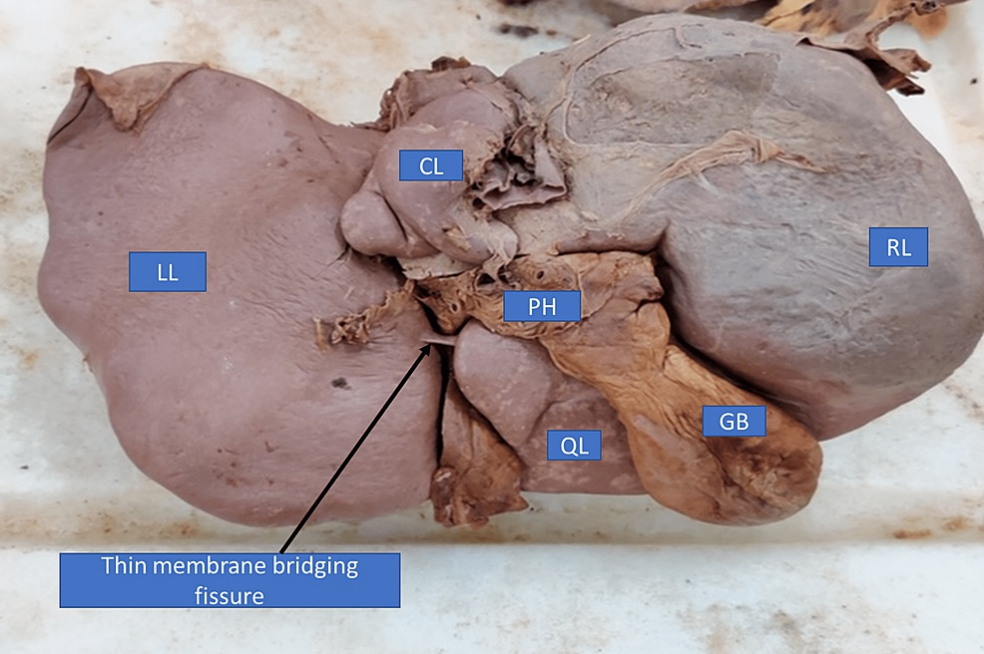
Liver with fissure covered by a thin membrane (inferior view) RL - right lobe; LL - left lobe; QL - quadrate lobe; CL - caudate lobe; GB - gall gladder; PH - porta hepatis

In this type of anatomical variant, instead of liver tissue, a thin membrane spanned the fissure connecting the left lobe and quadrate lobe. The fissure was complete extending up to the left end of porta hepatis and was present only on the posterior surface. This type of anatomical variantion in fissure was present in 6% of liver specimens (Figure 4, Table 1).

In all the livers of type II, III, and IV, fissure for ligamentum teres was approaching the inferior border.

## Discussion

Liver development starts after the third week of intrauterine life. It is a complex process controlled by various growth factors [11]. Consequently, during this developmental process, the liver can present several congenital anomalies, which various studies have documented. Variation in fissure for ligamentum teres hepatis was one anomaly that has been invariably reported by various authors but in different forms. In our study, we devised a classification based on our observation of variations following naked eye examination of the fissure, and various patterns of fissure for ligamentum teres hepatis were identified and accordingly classified into four types.

The most common type of variation in our study was type IV, accounting for 6%, in which the fissure was covered by a thin membrane connecting the left lobe and quadrate lobe. In a similar study, it was observed that in 10% of specimens, ligamentum teres were embedded in the groove, and it was covered by parenchymatous tissue of the liver from the side of the quadrate lobe like in our case [6] (Table 2). 

**Table 2:** Comparison of our study with various other studies

Authors	No. of specimens	Anomalous fissure for ligamentum teres hepatis	Type I anomaly	Type II anomaly	Type III anomaly	Type IV anomaly
Dave et al. [8] (Gujrat, India)	96	27 (28.1%)	1 (3.8%)	3 (11.1%)	NA	NA
Nayak et al. [9] (South India)	55	2 (3.6%)	NA	1 (1.81%)	1 (1.81%)	NA
Joshi et al [10] (South India)	90	30%	30%	NA	NA	NA
Saxena et al. [7] (Uttarakhand, India)	20	4 (20%)	3 (15%)	NA	1 (5%)	NA
Choy et al. [6] (Malaysia)	20	2 (10%)	NA	NA	NA	2 (10%)
Baruah et al. [15] (Assam, India)	30	1 (3.33%)	1 (3.33%)	NA	NA	NA
Present study (Uttar Pradesh)	100	15 (15%)	2 (2%)	3 (3%)	4 (4%)	6 (6%)

The second most common variety was type III, accounting for 4%. In this type, an incomplete fissure for ligamentum teres hepatis was present only on the visceral surface. A similar type of liver anomaly was reported in a study done at Government Medical College, Srinagar. In this study, 20% of the specimens showed anomalies in fissure for ligamentum teres, out of which 3.8% of specimens had an incomplete fissure like type III, whereas 11.1% of specimens had tunnels for ligamentum teres hepatis-like type I [7] (Table 2). A similar type of study was done in Gujrat, where 96 formalin-preserved livers were observed, out of which 27 livers (28.7%) had variant fissures for ligamentum teres. Out of these, 11.1% had fissures for ligamentum teres extending onto the anterior surface like type II anomalies, which was found to be 3% in our study, while on the other hand, 3.8% liver had tunnels for ligamentum teres hepatis and the tunnel was formed by pons hepatis [8] (Table 2).

Another study conducted on 55 liver specimens found one liver (1.81%) with a fissure present on the anterior surface as in type II, while the remaining one (1.81%) has an absence of fissure for ligamentum teres on the visceral surface, which was due to complete fusion of quadrate and left lobe of the liver [9] (Table 2). We could not find any liver with a complete absence of fissure in the visceral surface.

A study of 90 formalin-fixed livers done by Joshi et al. found 30% of livers had pons hepatis between the left lobe and quadrate lobe, like type I. In most cases, the pons was bridging the upper third of the fissure for ligamentum teres. In one case, the pons was present in the fissure's depth for ligamentum teres. In two cases, the pons completely bridged the fissure on the inferior surface, merging the left and the quadrate lobes [10] (Table 2). Type I anomalies having pons hepatis were the least dominant in our study, accounting for only 2%. A similar finding was reported in 1.25% of liver specimens studied at Medical College, Vadodara [12]. This was the most common anomaly reported by different authors, having a variable frequency, like Joshi et al. who found it in 30% of cases, and Patil et al. who found it in 10% of cases [10, 13] (Table 2). In their research, Aktan et al. discovered that 14.8% of the liver had fused left and quadrate lobes as a result of pons hepatis [14]. Similar to this, 3.33% of livers in a study conducted at Guwahati Medical College had their left and quadrate lobe fused across the fissure, whereas Arya et al. found 5% of livers with fused quadrate and left lobe [15,16] (Table 2). These variations will help anatomists understand the morphological variations of the liver and guide radiologists to do proper diagnosis without the confusion of any collections of fluid, metastatic deposits, or enlarged lymph nodes.

During fetal development, the left umbilical vein carries blood from the placenta to the liver, and communication is obtained between the vein and hepato-cardiac channel through ductus venosus [17]. This connection bypasses the sinusoidal plexus of the liver. After birth when the cord is clamped, there is a cessation of placental blood flow, as well as the beginning of respiration, which alters the oxygen tension. This initiates the obliteration of the vascular system. The umbilical arteries are closed by muscular contraction, followed by the umbilical veins and ductus venosus. The left umbilical vein gets obliterated by fibrosis and forms ligamentum teres hepatis, which is controlled by a variety of signaling factors [17]. This may also induce the surrounding hepatocytes to a variable degree to proliferate and form pons hepatis, leading to different types of anatomical variation in fissures for ligamentum teres hepatis [17]. This generates the further scope of this study to know the signaling factors responsible for the obliteration of the left umbilical vein to prove the probable hypothesis.

The primary limitation of the study was its restricted sample size, attributed to the unavailability of embalmed livers. This scarcity hindered the researchers' ability to gather a more extensive dataset for analysis. With a larger pool of samples, it would have been possible to establish a standardized classification system, enhancing the study's robustness and generalizability. Furthermore, to ascertain the types of cells present in the samples, additional validation measures could have been implemented. Specifically, the tissue obtained from the pons hepatis region could have been subjected to histopathological examination. This step would have provided valuable insights into the cellular composition and characteristics.

## Conclusions

The anatomical variation in fissure for ligamentum teres hepatis represents a commonly encountered anomaly in liver morphology. These variations hold clinical importance, as they may be mistaken for liver tumours and obstruct the sonographic evaluation of the umbilical segment of the left portal vein. By shedding light on the prevalence of these variations, the study underscores the importance of awareness among imaging specialists and surgeons. The insights from this research can aid in minimizing misunderstandings and subsequent misdiagnoses, while also facilitating the formulation of more effective surgical strategies. This study could be expanded through collaboration with radiological examinations of living individuals to understand the impact or significance of these variations.
